# Optic Disc Dose Comparison Between 125I and 103Pd Collaborative Ocular Melanoma Study (COMS) Plaques Based on Current Clinical Practice

**DOI:** 10.7759/cureus.15980

**Published:** 2021-06-28

**Authors:** Yongsook C Lee, Shih-Chi Lin, Yongbok Kim

**Affiliations:** 1 Radiation Oncology, Miami Cancer Institute, Miami, USA; 2 Radiation Oncology, Norwalk Hospital, Norwalk, USA; 3 Radiation Oncology, Duke University, Durham, USA

**Keywords:** optic disc dose, 125i, 103pd, coms plaques, ocular brachytherapy

## Abstract

Purpose

The purpose of this study is to compare optic disc dose (ODD) between ^125^I and ^103^Pd Collaborative Ocular Melanoma Study (COMS) plaques in ocular brachytherapy.

Methods

A previously validated in-house brachytherapy dose calculation program was used for ODD calculations. ODD was calculated as a function of tumor margin-to-optic disc distance (DT) up to 5 mm for various tumor basal dimensions (BDs), for a prescription depth of 5 mm, and for standard and notched COMS plaques loaded with ^125^I (model: IAI-125A) and ^103^Pd (model: IAPd-103A) seeds. ODD calculations were repeated for prescription depths from 2 mm to 10 mm in 1 mm intervals. A prescribed dose of 85 Gy (irradiation time: 120 hours) was normalized to each prescription depth. Dose conversion factors (DCFs) for each prescription depth were calculated by taking a ratio of [total reference air kerma (TRAK) per seed]_prescription_ _depth_ to [TRAK per seed]_5 mm_. ODD reduction by notched COMS plaques was calculated for each prescription depth by subtracting ODD for notched COMS plaques from ODD for standard COMS plaques.

Results

Trends of ODD as a function of DT for various BDs are similar between the two seed types in both standard and notched COMS plaques. However, due to the energy difference, there exists a transition distance (d_t_) for each BD in each plaque at which ODD for ^125^I COMS plaques equals that for ^103^Pd COMS plaques. For small BDs, at DT <d_t_, ODD for ^103^Pd COMS plaques is higher than that for ^125^I COMS plaques while at DT >d_t_, the opposite is observed. For the largest 1-3 BD(s), contrarily, d_t_ occurs within the tumor, and thus, ODD for ^125^I COMS plaquesis always higher. Trends of ODD reduction by notched COMS plaques as a function of DT for various BDs are the same for the two seed types except that maximum ODD reduction by ^103^Pd COMS notched plaques is larger. DCF increases with increasing prescription depth for both seed types.

Conclusions

There exist ODD differences between ^125^I and ^103^Pd COMS plaques and the differences depend on DT, BD, plaque size, and prescription depth.

## Introduction

In the mid-1980s, the Collaborative Ocular Melanoma Study (COMS) established standardized methods of ^125^I plaque brachytherapy for medium-sized (apical height from 2.5 mm to 10.0 mm and maximal tumor basal dimension (BD) of ≤16 mm) choroidal melanomas and conducted a prospective randomized clinical trial comparing ^125^I plaque brachytherapy with enucleation [1-3]. The COMS trial showed no significant survival benefit for enucleation [4,5]. Since then, plaque brachytherapy has become the most widely used eye- and vision-preserving treatment for uveal melanomas [6,7], and among multiple plaque designs, COMS plaques loaded with ^125^I seeds are the most common [2].

Although the COMS was restricted to the use of ^125^I seeds [1,3], ^103^Pd seeds have been used for plaque brachytherapy in North America [3]. Physical characteristics such as low average photon energies (28 keV for ^125^I and 21 keV for ^103^Pd) and relatively short half-lives (60 days for ^125^I and 17 days for ^103^Pd) have made both radionuclides suitable for plaque brachytherapy [2,3,8]. In 2009, Finger et al. reported improved local control and visual acuity outcomes with ^103^Pd plaques compared to using ^125^I plaques [9]. Their study included patients (92%) who were eligible for the COMS trial but refused to participate in the trial as well as those (8%) who were ineligible for the trial due to tumor size (3% of small-sized tumors and 5% of large-sized tumors). Several dosimetry studies have supported these clinical outcomes: with ^103^Pd plaques, the dose is increased within the tumor but decreased in most normal ocular structures when the equivalent dose is prescribed to the tumor apex [8-11]. This is due to the lower photon energy of ^103^Pd seeds resulting in a more rapid dose fall-off with distance compared with ^125^I seeds [9].

In our first simulation study, optic disc dose (ODD) as a function of distance from the tumor for standard COMS plaques loaded with ^125^I seeds was comprehensively investigated [12]. The investigation was made for three commercially available seed models (IsoAid Advantage IAI-125A, Best Industries 2301, and Bebig I25.S16) and for various prescription depths from 1 mm to 10 mm in 1 mm intervals. In the second simulation study, ODD reduction by notched COMS plaques with one and two seeds removed as a function of distance from the tumor was comprehensively investigated in comparison with ODD for standard COMS plaques [13]. The second study was performed for only one ^125^I seed model (IAI-125A) and for various prescription depths from 1 mm to 10 mm in 1 mm intervals. In both studies, based on our institutional practice, a prescribed dose of 85 Gy for an irradiation time of 168 hours (seven-day implant) was normalized to each prescription depth [12,13].

In this study, ODD as a function of distance from the tumor was compared between COMS plaques loaded with ^125^I seeds (model: IAI-125A, IsoAid LLC, Port Richey, FL) and COMS plaques loaded with ^103^Pd seeds (model: IAPd-103A, IsoAid LLC). Both standard COMS plaques and notched COMS plaques with one seed removed and with two seeds removed were included in this study. Following the recent American Brachytherapy Society (ABS) guidelines and clinical practice of institutions using ^103^Pd plaques [3,14], 85 Gy for an irradiation time of 120 hours (five-day implant) was prescribed to the tumor apex for both ^125^I and ^103^Pd COMS plaques. For this simulation study, an in-house brachytherapy dose calculation program, developed using MATLAB® software (vR2016a, MathWorks, Natick, MA) and previously validated against several commercial treatment planning systems [12], was used. Most clinics using COMS plaques follow the American Association of Physicists in Medicine Task Group (AAPM TG)-43 calculation without heterogeneity corrections. In this study, therefore, dose calculations were performed following the current clinical practice. Reporting ODD calculated using a more accurate dosimetry tool such as a Monte Carlo simulation is out of scope in this study.

## Materials and methods

ODD for ^125^I and ^103^Pd standard COMS plaques: prescription depth of 5 mm

**Figure 1:**
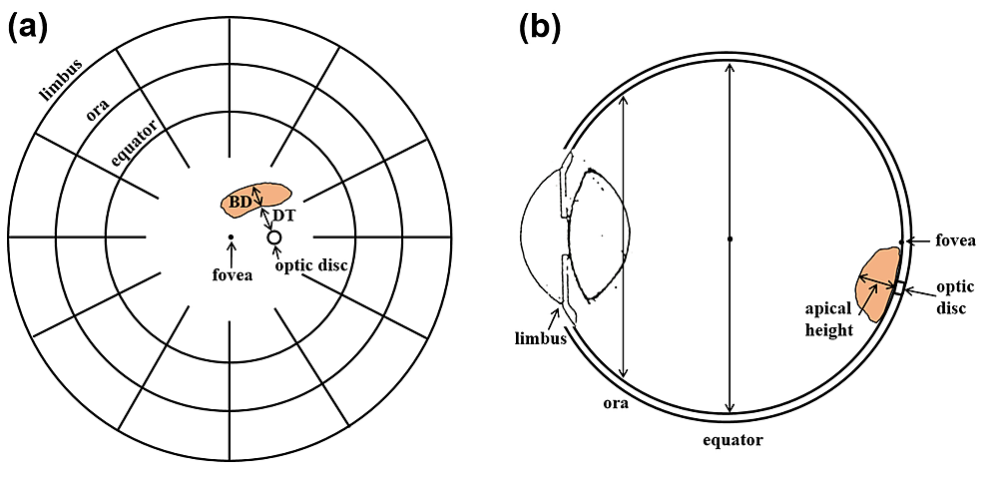
(a) A fundus diagram showing BD of the tumor and DT and (b) a cross-section of the eye showing the apical height of the tumor BD: basal dimension, DT: tumor margin-to-optic disc distance.

Using our validated brachytherapy dose calculation program [12], ODD for standard COMS plaques loaded with ^125^I seeds (model: IAI-125A) was calculated as a function of tumor margin (i.e., tumor edge)-to-optic disc distance (DT) up to 5 mm for various BDs. According to COMS protocols [1], BD of the tumor is defined as the basal dimension at the center in the direction from the optic disc (Figure 1(a)). The largest dimension of the tumor determines plaque size and is not always the same as BD. Based on the definition of medium-sized (maximal BD of ≤16 mm) tumors and a 2-3 mm margin around the tumor [2], calculations were performed for seven different-sized plaques from 10 mm to 22 mm in diameter in 2 mm increments. For each plaque, BDs up to (plaque diameter - 4) mm in 2 mm increments were included for calculations but small BDs <(plaque diameter - 4)/2 mm (even numbers) for 12 mm, 16 mm and 20 mm plaques or small BDs <(plaque diameter - 4)/2 - 1 mm (even numbers) for 10 mm, 14 mm, 18 mm, and 22 mm plaques were excluded. A prescribed dose of 85 Gy for an irradiation time of 120 hours was normalized to a central-axis depth of 5 mm. BD of the tumor and DT in a fundus diagram and apical height of the tumor in a cross-section of the eye are illustrated in Figure 1.

In the same fashion, ODD for standard COMS plaques loaded with ^103^Pd seeds (model: IAPd-103A) was calculated as a function of DT up to 5 mm for various BDs. Calculations were performed for seven plaques from 10 mm to 22 mm. The same dose (85 Gy) for the same irradiation time (120 hours) was prescribed to a central-axis depth of 5 mm.

ODD for ^125^I and ^103^Pd notched COMS plaques: prescription depth of 5 mm

**Figure 2:**
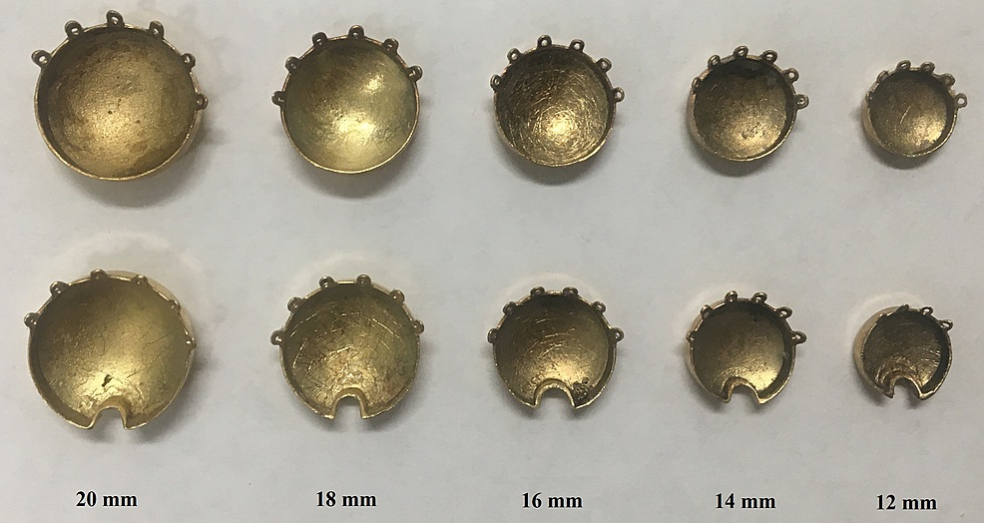
A photo of standard (top row) and notched (bottom row) COMS plaques with diameters from 12 mm to 20 mm in 2 mm increments COMS: Collaborative Ocular Melanoma Study.

Notched plaques are used to accommodate the optic disc when the tumor is adjacent to it (Figure 2). Notched COMS plaques were designed to remove one seed but two seeds can be removed. In this study, therefore, two cases (notched COMS plaques with one seed removed and with two seeds removed) were considered. For one seed removal, six different-sized notched plaques from 12 mm to 22 mm in diameter in 2 mm increments were included. A 10 mm plaque was excluded because a notched plaque design is not available for this plaque. Removed seed position numbers in Figure 1 of the AAPM TG-129 report were 3, 4, 4, 5, 5, and 5 for 12 mm, 14 mm, 16 mm, 18 mm, 20 mm, and 22 mm plaques, respectively [2]. For two seeds removal, five different-sized notched plaques from 14 mm to 22 mm were included. A 12 mm plaque was excluded because its seed configuration does not allow for two seeds removal. Removed seed position numbers were (4, 9), (4, 10), (5, 12), (5, 13), and (5, 12) for 14 mm, 16 mm, 18 mm, 20 mm, and 22 mm plaques, respectively [2].

ODD for notched COMS plaques loaded with ^125^I seeds (model: IAI-125A) was calculated as a function of DT up to 5 mm for various BDs. Calculations were performed for one seed removal (plaques from 12 mm to 22 mm) and two seeds removal (plaques from 14 mm to 22 mm). A BD range for each notched COMS plaque was the same as for the corresponding standard COMS plaque. After seed(s) were removed from standard COMS plaques, 85 Gy for an irradiation time of 120 hours was normalized to a central-axis depth of 5 mm.

In the same fashion, ODD for notched COMS plaques loaded with ^103^Pd seeds (model: IAPd-103A) was calculated as a function of DT up to 5 mm for various BDs. Calculations were performed for one seed removal (plaques from 12 mm to 22 mm) and two seeds removal (plaques from 14 mm to 22 mm). The same dose (85 Gy) for the same irradiation time (120 hours) was prescribed to a central-axis depth of 5 mm after seed(s) were removed.

Dose conversion factors for different prescription depths

Dose conversion factors (DCFs) for different prescription depths were generated for ^125^I standard COMS plaques and ^103^Pd standard COMS plaques. ODD calculations for ^125^I standard COMS plaques were repeated for various central-axis depths from 2 mm to 10 mm in 1 mm intervals based on the definition of medium-sized tumors (apical height from 2.5 mm to 10.0 mm). For each prescription depth, calculations were performed for seven different-sized plaques from 10 mm to 22 mm in diameter. A prescribed dose of 85 Gy for an irradiation time of 120 hours was normalized to each depth. For each depth and each plaque, total reference air kerma (TRAK = air kerma strength Sk × irradiation time, unit: μGym^2^) per seed was calculated. Then, DCF for each depth and each plaque was obtained by calculating a ratio of [TRAK per seed]_prescription depth_/[TRAK per seed]_5 mm _where a prescription depth ranges from 2 mm to 10 mm in 1 mm intervals. Likewise, DCFs for ^103^Pd standard COMS plaques were obtained. DCFs will allow for estimating ODD at any prescription depth by multiplying ODD at 5 mm by DCF at a depth of interest.

In addition, DCFs for different prescription depths were generated for ^125^I notched COMS plaques and ^103^Pd notched COMS plaques. ODD calculations for ^125^I notched COMS plaques were repeated for various prescription depths from 2 mm to 10 mm in 1 mm intervals. For each prescription depth, calculations were performed for one seed removal (plaques from 12 mm to 22 mm) and two seeds removal (plaques from 14 mm to 22 mm). The same dose (85 Gy) for the same irradiation time (120 hours) was prescribed to each depth after seed(s) were removed. TRAK per seed for each depth and each plaque was calculated and then DCF for each depth and each plaque was obtained in the same way as for ^125^I standard COMS plaques. Likewise, DCFs for ^103^Pd notched COMS plaques were obtained.

ODD reduction by ^125^I and ^103^Pd notched COMS plaques

From ODDs for ^125^I standard COMS plaques and ^125^I notched COMS plaques, ODD reduction by ^125^I notched COMS plaques was calculated as a function of DT for various BDs. Absolute ODD reduction, $ODD{reduction}^{Abs} (Gy)$, was computed using equation (1). Calculations were performed for prescription depths from 2 mm to 10 mm in 1 mm intervals and for one seed removal (plaques from 12 mm to 22 mm) and two seeds removal (plaques from 14 mm to 22 mm).

 $ODD{reduction}^{Abs} (Gy) = ODD{standard}-ODD{notched}$ (1)

ODD reduction by ^103^Pd notched COMS plaques was also calculated as a function of DT for various BDs. $ODD{reduction}^{Abs} (Gy)$ was computed for prescription depths from 2 mm to 10 mm and for one seed removal (plaques from 12 mm to 22 mm) and two seeds removal (plaques from 14 mm to 22 mm) using equation (1).

## Results

ODD comparison between ^125^I and ^103^Pd standard COMS plaques: prescription depth of 5 mm

**Figure 3:**
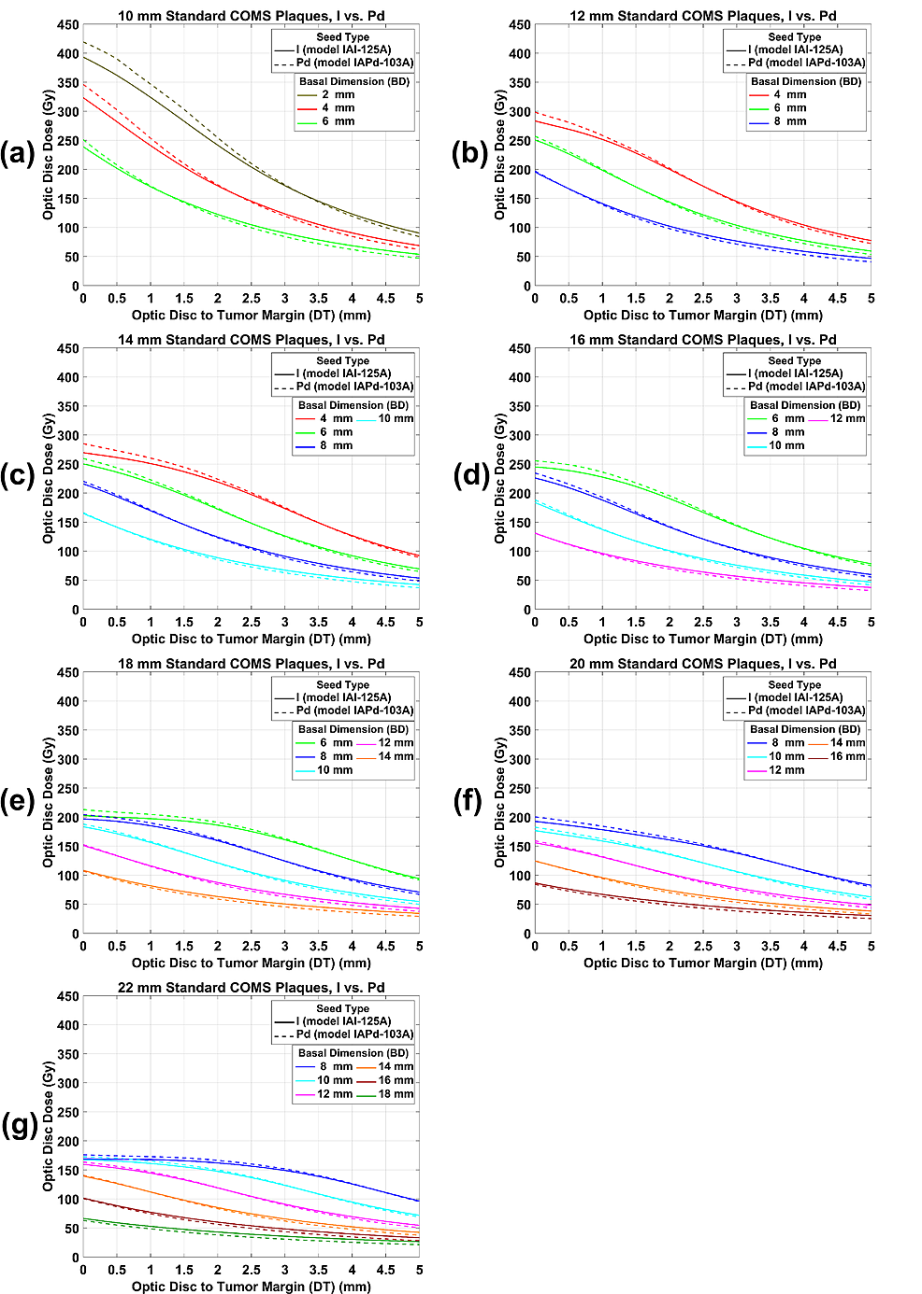
(a)-(g) An ODD comparison between 125I and 103Pd standard COMS plaques as a function of DT for various BDs. A prescribed dose of 85 Gy was normalized to a central-axis depth of 5 mm. ODD: optic disc dose, COMS: Collaborative Ocular Melanoma Study, DT: tumor margin-to-optic disc distance, BD: basal dimension.

Figure 3(a)-3(g) shows plots of ODD for ^125^I versus ^103^Pd standard COMS plaques as a function of DT for various BDs when 85 Gy was prescribed to a central-axis depth of 5 mm. Trends of ODD for ^125^I standard COMS plaques are similar to those for ^103^Pd standard COMS plaques. ODD for both seed types usually decreases as DT and BD increase. On the other hand, there are differences in ODD between ^125^I and ^103^Pd standard COMS plaques. Compared with that for ^125^I plaques, ODD for ^103^Pd plaques is higher, becomes the same, and then is lower as DT increases. Therefore, for each BD (excluding the largest 1-2 BDs) in each plaque, there exists a transition distance (d_t_) at which ODD for ^103^Pd standard plaques is equivalent to ODD for ^125^I standard plaques. For each plaque, d_t_ becomes closer to the optic disc by about 1 mm with increasing BD by 2 mm. For the largest 1-2 BDs in plaques ≥18 mm, however, d_t_ occurs within the tumor, and as a result, ODD for ^125^I standard plaques is always higher than ODD for ^103^Pd standard plaques.

ODD comparison between ^125^I and ^103^Pd notched COMS plaques: prescription depth of 5 mm

**Figure 4:**
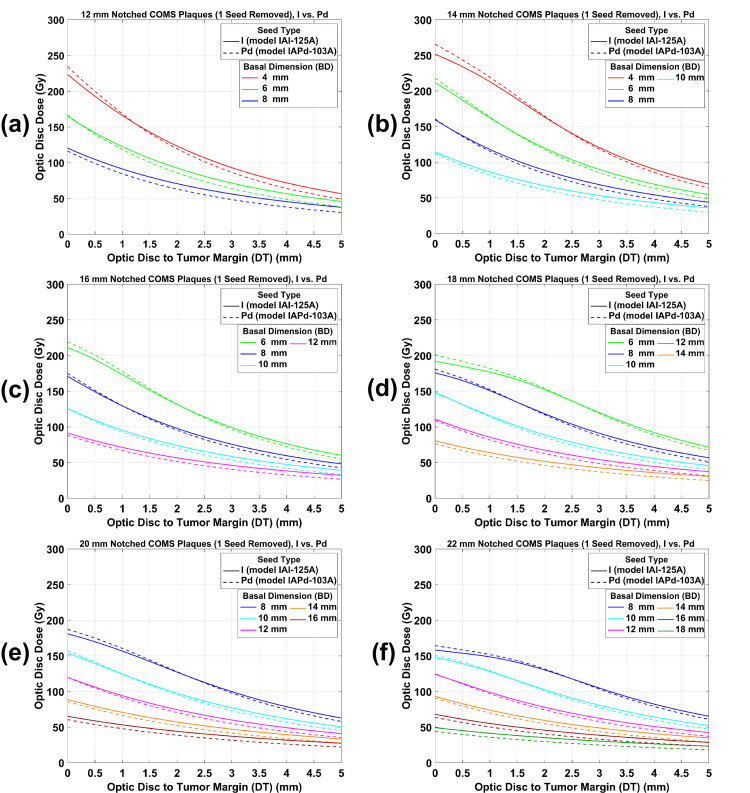
(a)-(f) An ODD comparison between 125I and 103Pd notched COMS plaques with one seed removed as a function of DT for various BDs. A prescribed dose of 85 Gy was normalized to a central-axis depth of 5 mm after one seed was removed from standard COMS plaques. ODD: optic disc dose, COMS: Collaborative Ocular Melanoma Study, DT: tumor margin-to-optic disc distance, BD: basal dimension.

Figure 4(a)-4(f) shows plots of ODD for ^125^I versus ^103^Pd notched COMS plaques with one seed removed as a function of DT for various BDs when 85 Gy was prescribed to a central-axis depth of 5 mm. As for standard COMS plaques, trends of ODD for ^125^I notched COMS plaques are similar to those for ^103^Pd notched COMS plaques: ODD for both seed types usually decreases as DT and BD increase. Also, trends of an ODD comparison between the two seed types for notched plaques are similar to those for standard plaques: d_t_, at which ODD for ^103^Pd notched plaques equals ODD for ^125^I notched plaques, exists for each BD (excluding the largest 1-3 BDs) in each plaque and ODD for ^103^Pd notched plaques is higher than that for ^125^I notched plaques at DT <d_t_ but the opposite is observed at DT >d_t_. For the largest 1-3 BDs, ODD for ^125^I notched plaques is always higher.

**Figure 5:**
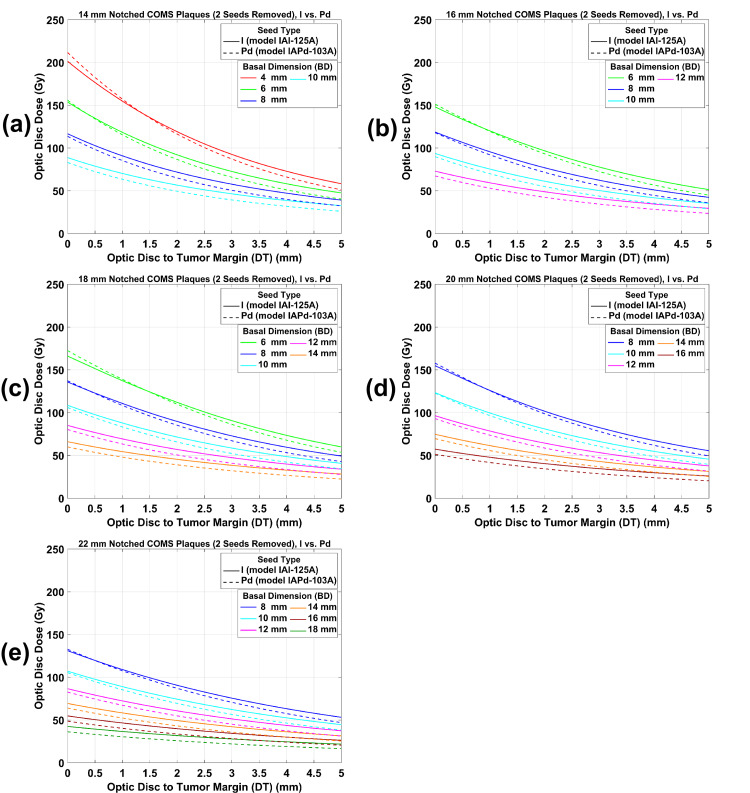
(a)-(e) An ODD comparison between 125I and 103Pd notched COMS plaques with two seeds removed as a function of DT for various BDs. A prescribed dose of 85 Gy was normalized to a central-axis depth of 5 mm after two seeds were removed from standard COMS plaques. ODD: optic disc dose, COMS: Collaborative Ocular Melanoma Study, DT: tumor margin-to-optic disc distance, BD: basal dimension.

ODD for ^125^I versus ^103^Pd notched COMS plaques with two seeds removed as a function of DT for various BDs are plotted in Figure 5(a)-5(e). Trends of an ODD comparison between ^125^I and ^103^Pd notched plaques with two seeds removed are similar to those with one seed removed but ODD for two seeds removal is lower.

ODD reduction comparison between ^125^I and ^103^Pd notched COMS plaques: prescription depth of 5 mm

**Figure 6:**
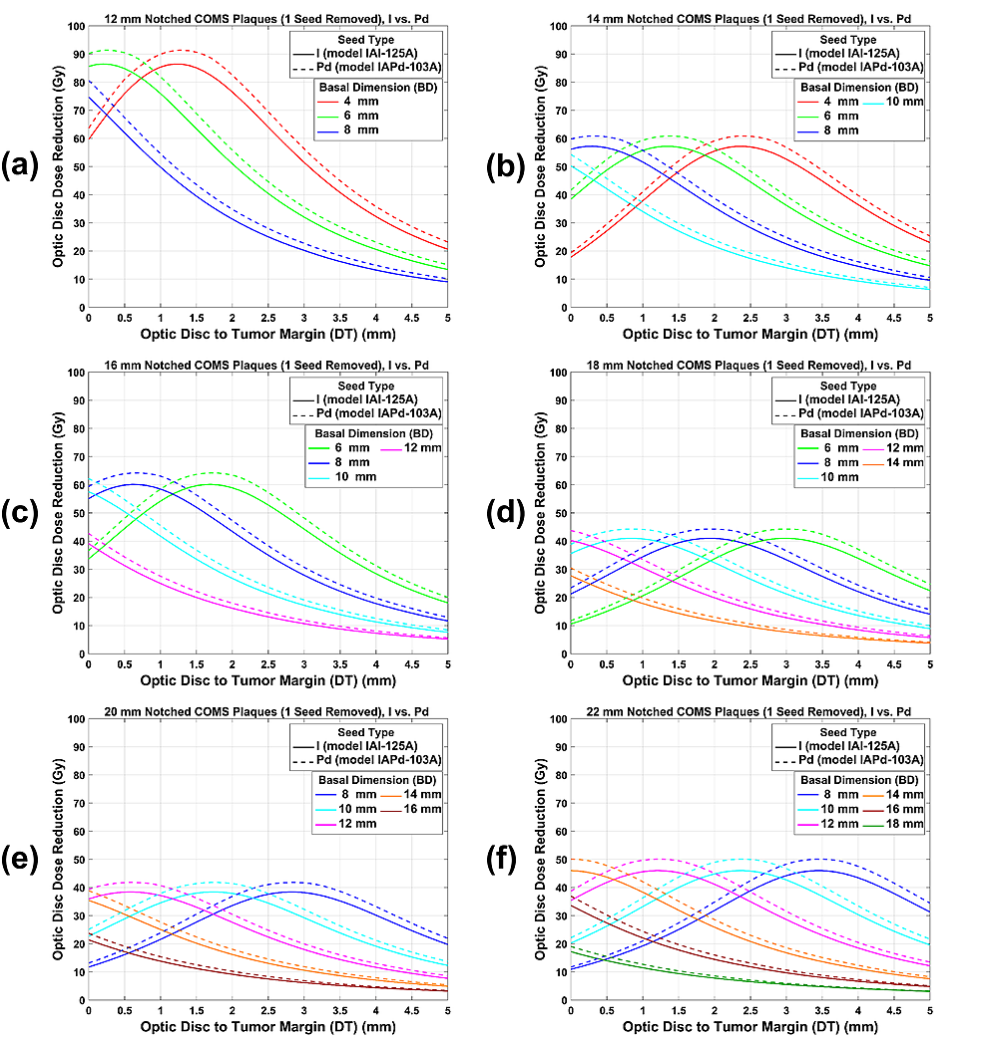
(a)-(f) An ODD reduction comparison between 125I and 103Pd notched COMS plaques with one seed removed as a function of DT for various BDs. A prescribed dose of 85 Gy was normalized to a central-axis depth of 5 mm before the ODD reduction was calculated. ODD: optic disc dose, COMS: Collaborative Ocular Melanoma Study, DT: tumor margin-to-optic disc distance, BD: basal dimension.

Figure 6(a)-6(f) shows plots of ODD reduction by ^125^I versus ^103^Pd notched COMS plaques with one seed removed as a function of DT for various BDs when 85 Gy was prescribed to a central-axis depth of 5 mm. These plots were obtained by subtracting plots in Figure 4(a)-4(f) from plots in Figure 3(b)-3(g). Trends for ^125^I notched plaques are the same as those for ^103^Pd notched plaques except that ODD reduction is larger with ^103^Pd notched plaques. ODD reduction increases with increasing DT, reaches the maximum value ($MaxODD{reduction}^{Abs} (Gy)$), and then decreases with increasing DT. Contrarily, for the largest 1-2 BDs, ODD reduction decreases continuously with increasing DT and thus, $MaxODD{reduction}^{Abs} (Gy)$ occurs at 0 mm. Excluding the largest 1-2 BDs, differences of $MaxODD{reduction}^{Abs} (Gy)$ between ^103^Pd and ^125^I notched plaques are 5.1 Gy, 3.8 Gy, 4.3 Gy, 3.4 Gy, 3.5 Gy, and 4.5 Gy for 12 mm, 14 mm, 16 mm, 18 mm, 20 mm, and 22 mm plaques, respectively.

**Figure 7:**
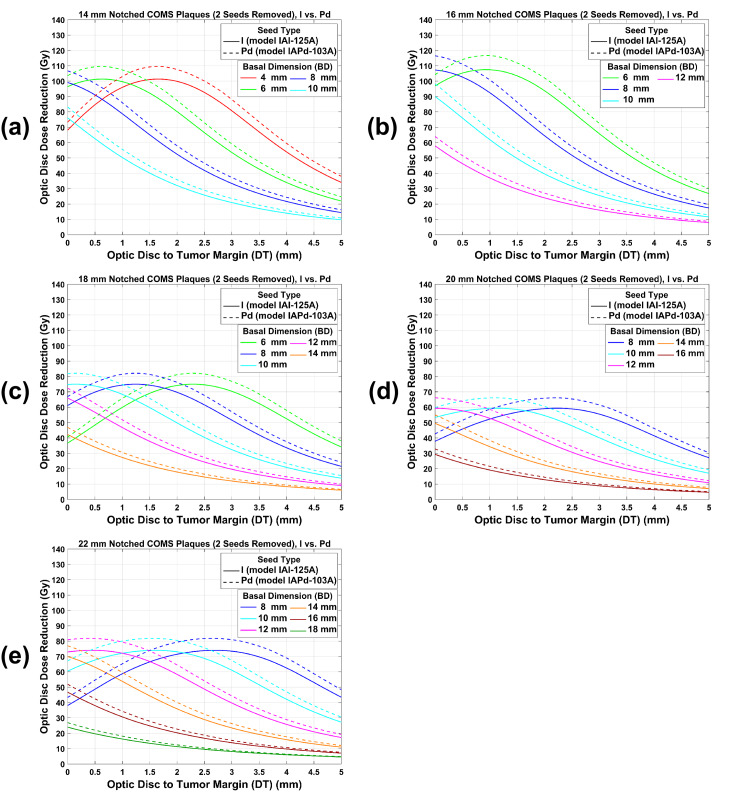
(a)-(e) An ODD reduction comparison between 125I and 103Pd notched COMS plaques with two seeds removed as a function of DT for various BDs. A prescribed dose of 85 Gy was normalized to a central-axis depth of 5 mm before the ODD reduction was calculated. ODD: optic disc dose, COMS: Collaborative Ocular Melanoma Study, DT: tumor margin-to-optic disc distance, BD: basal dimension.

ODD reduction by ^125^I versus ^103^Pd notched COMS plaques with two seeds removed as a function of DT for various BDs is plotted in Figure 7(a)-7(e). Trends of ODD reduction comparison between ^125^I and ^103^Pd notched plaques with two seeds removed are similar to those with one seed removed but an ODD reduction for ^125^I and ^103^Pd notched plaques is larger and ODD reduction differences between ^125^I and ^103^Pd notched plaques are also larger for two seeds removal.

DCF comparison between ^125^I and ^103^Pd COMS plaques

Table 1 presents a comparison of DCFs for prescription depths from 2 mm to 10 mm between ^125^I and ^103^Pd standard COMS plaques. DCF increases with increasing depth in each plaque for both seed types. However, the increase is more drastic with ^103^Pd plaques. As a result, at a depth of <5 mm, DCFs for ^103^Pd plaques are smaller than those for ^125^I plaques and absolute differences between the two seed types range from 0.01 to 0.03. On the other hand, at a depth of >5 mm, DCFs for ^103^Pd plaques are larger than those for ^125^I plaques and absolute differences are much larger, increasing with increasing depth. For example, the largest absolute difference for the 10 mm plaque is 0.45 (3.29 for ^103^Pd plaques versus 2.84 for ^125^I plaques) at a prescription depth of 10 mm.

**Table 1:** A comparison of DCFs (ratios of TRAK per seed) between 125I (model: IAI-125A) and 103Pd (model: IAPd-103A) standard COMS plaques. The data were normalized to those for a prescription depth of 5 mm. DCF: dose conversion factor, TRAK: total reference air kerma, COMS: Collaborative Ocular Melanoma Study.

Prescription depth (mm)	Plaque size in diameter (mm)													
	10		12		14		16		18		20		22	
	^125^I	^103^Pd	^125^I	^103^Pd	^125^I	^103^Pd	^125^I	^103^Pd	^125^I	^103^Pd	^125^I	^103^Pd	^125^I	^103^Pd
2	0.41	0.39	0.45	0.43	0.48	0.46	0.52	0.50	0.55	0.53	0.58	0.55	0.59	0.56
3	0.57	0.55	0.60	0.58	0.63	0.60	0.66	0.63	0.68	0.66	0.70	0.67	0.71	0.68
4	0.77	0.75	0.78	0.77	0.80	0.78	0.81	0.80	0.83	0.81	0.84	0.82	0.85	0.83
5	1.00	1.00	1.00	1.00	1.00	1.00	1.00	1.00	1.00	1.00	1.00	1.00	1.00	1.00
6	1.27	1.31	1.25	1.29	1.23	1.27	1.22	1.25	1.20	1.23	1.18	1.21	1.18	1.20
7	1.59	1.68	1.54	1.63	1.50	1.59	1.47	1.55	1.43	1.50	1.40	1.46	1.38	1.44
8	1.95	2.13	1.88	2.05	1.82	1.97	1.76	1.91	1.69	1.82	1.64	1.76	1.61	1.72
9	2.37	2.66	2.27	2.54	2.18	2.43	2.10	2.33	2.00	2.21	1.92	2.12	1.87	2.06
10	2.84	3.29	2.71	3.13	2.59	2.98	2.48	2.84	2.34	2.67	2.24	2.54	2.17	2.45

Table 2 presents a comparison of DCFs for prescription depths from 2 mm to 10 mm between ^125^I and ^103^Pd notched COMS plaques with one seed removed. Trends of DCFs for notched plaques are the same as those for standard plaques: DCF increases with increasing depth in each plaque for both seed types. DCFs for ^103^Pd notched plaques are smaller than those for ^125^I plaques at a depth of <5 mm, whereas the opposite is observed at a depth of >5 mm.

**Table 2:** A comparison of DCFs (ratios of TRAK per seed) between 125I (model: IAI-125A) and 103Pd (model: IAPd-103A) notched COMS plaques with one seed removed. The data were normalized to those for a prescription depth of 5 mm. DCF: dose conversion factor, TRAK: total reference air kerma, COMS: Collaborative Ocular Melanoma Study.

Prescription depth (mm)	Plaque size in diameter (mm)											
	12		14		16		18		20		22	
	^125^I	^103^Pd	^125^I	^103^Pd	^125^I	^103^Pd	^125^I	^103^Pd	^125^I	^103^Pd	^125^I	^103^Pd
2	0.45	0.42	0.48	0.45	0.52	0.49	0.55	0.52	0.57	0.55	0.58	0.55
3	0.60	0.57	0.62	0.60	0.65	0.63	0.68	0.65	0.70	0.67	0.70	0.68
4	0.78	0.76	0.80	0.78	0.81	0.79	0.83	0.81	0.84	0.82	0.84	0.83
5	1.00	1.00	1.00	1.00	1.00	1.00	1.00	1.00	1.00	1.00	1.00	1.00
6	1.26	1.29	1.24	1.27	1.22	1.25	1.20	1.23	1.19	1.21	1.18	1.21
7	1.55	1.64	1.51	1.60	1.48	1.56	1.43	1.51	1.40	1.47	1.38	1.45
8	1.89	2.06	1.83	1.99	1.77	1.92	1.70	1.83	1.65	1.77	1.62	1.74
9	2.28	2.56	2.20	2.45	2.11	2.35	2.01	2.22	1.94	2.13	1.89	2.08
10	2.73	3.15	2.61	3.00	2.50	2.86	2.36	2.69	2.26	2.56	2.19	2.48

A comparison of DCFs for prescription depths from 2 mm to 10 mm between ^125^I and ^103^Pd notched COMS plaques with two seeds removed is shown in Table 3. Trends of DCFs for one seed removal are the same as those for two seeds removal.

**Table 3:** A comparison of DCFs (ratios of TRAK per seed) between 125I (model: IAI-125A) and 103Pd (model: IAPd-103A) notched COMS plaques with two seeds removed. The data were normalized to those for a prescription depth of 5 mm. DCF: dose conversion factor, TRAK: total reference air kerma, COMS: Collaborative Ocular Melanoma Study.

Prescription depth (mm)	Plaque size in diameter (mm)									
	14		16		18		20		22	
	^125^I	^103^Pd	^125^I	^103^Pd	^125^I	^103^Pd	^125^I	^103^Pd	^125^I	^103^Pd
2	0.48	0.45	0.52	0.49	0.55	0.52	0.57	0.54	0.57	0.54
3	0.62	0.60	0.65	0.63	0.67	0.65	0.69	0.67	0.70	0.67
4	0.80	0.78	0.81	0.79	0.83	0.81	0.84	0.82	0.84	0.82
5	1.00	1.00	1.00	1.00	1.00	1.00	1.00	1.00	1.00	1.00
6	1.24	1.27	1.22	1.25	1.20	1.23	1.19	1.22	1.18	1.21
7	1.51	1.60	1.47	1.55	1.43	1.51	1.40	1.47	1.39	1.45
8	1.83	1.98	1.77	1.91	1.70	1.84	1.65	1.78	1.62	1.74
9	2.19	2.45	2.11	2.34	2.01	2.23	1.94	2.14	1.89	2.08
10	2.60	3.00	2.49	2.85	2.36	2.69	2.26	2.56	2.20	2.48

Estimation of ODD: clinical application of this study

For a clinical example (BD = 6 mm, DT = 2 mm, and apical height = 4 mm), a practical application of the results (figures and tables) from this study is shown in Table 4. A prescribed dose is 85 Gy and a 16 mm COMS plaque is selected. Depending on the seed type (^125^I versus ^103^Pd), COMS plaque type (standard versus notched), and prescription depth (COMS protocols versus ABS guidelines), 12 scenarios are possible, and ODD can be estimated for each scenario (Table 4). From the 12 scenarios, the following trends are observed. First, ODD for standard plaques is higher than that for notched plaques (scenarios #1 versus #2 and #3; scenarios #4 versus #5 and #6; scenarios #7 versus #8 and #9; scenarios #10 versus #11 and #12) for the same seed type and for the same prescription depth. Second, ODD for a prescription depth of 5 mm (COMS protocols) is higher than for a prescription depth of 4 mm (i.e., tumor apex; ABS guidelines, scenarios #1-#6 versus #7-#12). Third, when 85Gy was prescribed at 5 mm, ODD (195.48 Gy) for the ^103^Pd standard plaque is higher than that (190.43 Gy) for the ^125^I standard plaque. On the other hand, ODD (131.89 Gy) for the ^103^Pd notched plaque with one seed removed is similar to that (131.52 Gy) for the ^125^I notched plaque with one seed removed and ODD (93.27 Gy) for the ^103^Pd notched plaque with two seeds removed is lower than that (96.71 Gy) for the ^125^I notched plaque with two seeds removed. Fourth, when 85Gy was prescribed at the tumor apex (i.e., 4 mm), ODD (156.38 Gy) for the ^103^Pd standard plaque is higher than that (154.25 Gy) for the ^125^I standard plaque. The opposite trend is observed for notched plaques (106.53 Gy for ^125^I notched plaque with one seed removed versus 104.19 Gy for ^103^Pd notched plaque with one seed removed; 78.34 Gy for ^125^I notched plaque with two seeds removed versus 73.68 Gy for ^103^Pd notched plaque with two seeds removed). Therefore, among the 12 scenarios, the ^103^Pd standard plaque prescribed at 5 mm and the ^103^Pd notched plaque with two seeds removed prescribed at 4 mm give the highest ODD (195.48 Gy for scenario #4) and the lowest ODD (73.68 Gy for scenario #12), respectively, and the dose difference between these two scenarios is 121.80 Gy (143.29% of the prescribed dose).

**Table 4:** Twelve possible scenarios for a clinical example (BD = 6 mm, DT = 2 mm, and apical height = 4 mm in 16 mm COMS plaques) and corresponding estimated ODDs BD: basal dimension, DT: tumor margin-to-optic disc distance, COMS: Collaborative Ocular Melanoma Study, ODD: optic disc dose.

Scenario #	Seed type	COMS plaque-type	Prescription depth (mm)	Dose conversion factor relative to the depth of 5 mm	Estimated ODD (Gy)	Absolute dose reduction from standard plaque (Gy)	Relative dose reduction from standard plaque (%)	Reference
1	^125^I	Standard	5	1.00	190.43	Unavailable	Unavailable	Figure 3(d)
2	^125^I	Notched with one seed removed	5	1.00	131.52	58.91	30.94	Figure 4(c)
3	^125^I	Notched with two seeds removed	5	1.00	96.71	93.72	49.21	Figure 5(b)
4	^103^Pd	Standard	5	1.00	195.48	Unavailable	Unavailable	Figure 3(d)
5	^103^Pd	Notched with one seed removed	5	1.00	131.89	63.59	32.53	Figure 4(c)
6	^103^Pd	Notched with two seeds removed	5	1.00	93.27	102.21	52.29	Figure 5(b)
7	^125^I	Standard	4	0.81	154.25	Unavailable	Unavailable	Figure 3(d) and Table 1
8	^125^I	Notched with one seed removed	4	0.81	106.53	47.72	30.94	Figure 4(c) and Table 2
9	^125^I	Notched with two seeds removed	4	0.81	78.34	75.91	49.21	Figure 5(b) and Table 3
10	^103^Pd	Standard	4	0.80	156.38	Unavailable	Unavailable	Figure 3(d) and Table 1
11	^103^Pd	Notched with one seed removed	4	0.79	104.19	52.19	33.37	Figure 4(c) and Table 2
12	^103^Pd	Notched with two seeds removed	4	0.79	73.68	82.70	52.88	Figure 5(b) and Table 4

## Discussion

This study has demonstrated that ODD has a dependence on DT, BD, plaque size, and prescription depth, and the trends of ODD for ^125^I standard and notched COMS plaques are similar to those for ^103^Pd standard and notched COMS plaques, respectively (Figures 3-5). ODD usually decreases with increasing DT mainly due to the inverse square law. For the same DT in each plaque, ODD decreases with increasing BD because the distance from plaque to optic disc increases with increasing BD. Since the diameter of the plaque, the number of seeds, and seed configurations depend on the plaque [2], ODD varies with plaque size. ODD increases with increasing prescription depth because a deeper depth requires greater TRAK per seed (Tables 1-3).

Despite the aforementioned similarities, there are differences in ODD between ^125^I and ^103^Pd COMS plaques (Figures 3-5). The differences result from the difference in average photon energy between the two seed types (28 keV for ^125^I seeds versus 21 keV for ^103^Pd seeds) which leads to a more drastic decrease of relative dose with distance for ^103^Pd seeds than for ^125^I seeds [9]. Therefore, ODD for ^103^Pd plaques is higher at relatively short DT and becomes lower at longer DT than ODD for ^125^I plaques. As a result, there exists the transition point (i.e., d_t_) at which ODD for ^103^Pd plaques is the same as that for ^125^I plaques. For small BDs in each plaque, d_t_ becomes closer to the optic disc with increasing BD because the region of drastic dose fall-off within the tumor increases as BD increases.

ODD reduction by notched COMS plaques has unique trends and there are similarities and differences in ODD reduction between ^125^I and ^103^Pd notched COMS plaques (Figures 6 and 7). Reasons for the unique trends of ODD reduction are detailed in Lee et al. [13]. Similarities in ODD reduction between the two seed types can be explained by the reasons. Differences in ODD reduction between the two seed types result from the energy difference. Because of the lower energy and more rapid dose fall-off with increasing DT, ODD reduction by ^103^Pd notched plaques is larger than that by ^125^I notched plaques, and therefore, $MaxODD{reduction}^{Abs} (Gy)$ for ^103^Pd notched plaques is larger (Figures 6 and 7). However, for each plaque, there is no difference in DT_maxD_ (DT at which $MaxODD{reduction}^{Abs} (Gy)$ occurs) between the two seed types because the number of seeds and seed configurations in each plaque is the same for both seed types.

There are similarities and differences in DCFs between ^125^I and ^103^Pd COMS plaques (Tables 1-3). DCF for both seed types increases with increasing prescription depth because a deeper depth requires greater TRAK per seed (Tables 1-3). Since dose fall-off along the central axis of the plaque is more rapid with ^103^Pd plaques, at prescription depths shallower than 5 mm, the central-axis dose for ^103^Pd plaques is higher than that for ^125^I plaques, and thus, smaller DCFs are required with ^103^Pd plaques. At prescription depths deeper than 5 mm, the central-axis dose for ^103^Pd plaques becomes lower, and thus, larger DCFs are required with ^103^Pd plaques. As a result, DCF for ^103^Pd plaques increases more rapidly with increasing prescription depth than that for ^125^I plaques. Both standard and notched COMS plaques have these trends.

Data for the other prescription depths can be obtained from the data provided in this study. In this study, ODD for both seed types and ODD reduction by notched plaques for both seed types were presented only for a prescription depth of 5 mm. Yet, corresponding data for the other prescription depths would be different. From ODD (Figures 3-5) for a depth of 5 mm and DCFs (Tables 1-3) for depths of 2-4 mm and 6-10 mm, one can calculate ODD for any prescription depth and compare ODD between ^125^I and ^103^Pd COMS plaques.

The clinical example comparing ODDs among 12 different scenarios would help the clinician choose the best option to minimize ODD. As shown in Table 4, for tumors with apical height <5 mm, seed type (^125^I or ^103^Pd), COMS plaque type (standard or notched), and prescription depth (5 mm or tumor apex) affect ODD. ODD for ^103^Pd seeds is not always lower than that for ^125^I seeds, contradicting the study by Finger et al. [9]. ODD for ^125^I seeds can be lower when the tumor is closer to the optic disc than d_t_ (scenarios #1 versus #4). Notched plaques are usually beneficial in reducing ODD. Prescribing to a shallower depth is also beneficial in reducing ODD. For tumors with apical height ≥5 mm, however, a prescription depth is always the tumor apex and thus, a prescription depth would not be a parameter to reduce ODD.

One limitation of this study is that heterogeneity corrections were not taken into account. It has been reported that dose attenuated in the silastic insert and gold plaque is significant (≥10%) [15]. Hence, the ODD presented in this study would be overestimating. AAPM TG 129 and AAPM TG 221 recommend the TG-43 calculations along with heterogeneity corrections [2,16]. A future study using a more accurate dose calculation tool such as Plaque Simulator (Eye Physics LLC, Los Alamitos, CA) or another Monte Carlo simulation would warrant accurate ODD.

## Conclusions

This study has demonstrated that ODD has dependence on DT, BD, plaque size, plaque type, prescription depth, and seed type. Trends of ODD as a function of DT for various BDs are similar between ^125^I and ^103^Pd seeds for both standard and notched COMS plaques. However, due to the energy difference between the two seed types resulting in different relative dose variations with distance, there exists a transition distance (d_t_) for each BD in each plaque at which ODD for ^125^I plaques equals that for ^103^Pd plaques. For small BDs, at DT <d_t_, ODD for ^103^Pd plaques is higher than that for ^125^I plaques while at DT >d_t_, the opposite is observed. For the largest 1-3 BDs, contrarily, d_t_ exists within the tumor and thus, ODD for ^125^I plaques is always higher than that for ^103^Pd plaques. Trends of ODD reduction by notched plaques as a function of DT for various BDs are the same for both seed types except that $MaxODD{reduction}^{Abs} (Gy)$ by ^103^Pd notched plaques is larger than that by ^125^I notched plaques. ODD and DCF increase with increasing prescription depth for both seed types but DCF for ^103^Pd plaques increases more drastically than for ^125^I plaques.
